# Cyclo(l-tyrosyl-l-tryptophanyl) dimethylformamide solvate

**DOI:** 10.1107/S1600536808000640

**Published:** 2008-01-11

**Authors:** Carl Henrik Görbitz, Lars Male Hartviksen

**Affiliations:** a Department of Chemistry, University of Oslo, PO Box 1033 Blindern, N-0315 Oslo, Norway

## Abstract

The structure of the title compound [systematic name: (3*S*,6*S*)-3-(4-hydroxy­benz­yl)-6-(1*H*-indol-3-ylmeth­yl)piperazine-2,5-dione dimethyl­formamide solvate], C_20_H_19_N_3_O_3_·C_3_H_7_NO, contains hydrogen-bonded tapes typical for diketopiperazines. The structure is stabilized by strong inter­molecular inter­actions of the types O—H⋯O and N—H⋯O involving the dipeptide and the solvent mol­ecules. The absolute configuration was known from the starting materials.

## Related literature

For related structures, see: Morris *et al.* (1974 ▶); Grant *et al.* (1999 ▶); Suguna *et al.* (1984 ▶); Lin & Webb (1973 ▶); Razak *et al.*, (2000 ▶); Luo & Palmore (2002 ▶); Görbitz (1987 ▶); Görbitz & Hartviksen (2006 ▶). Solvent inclusion: Görbitz & Hersleth (2000 ▶). Cambridge Structural Database: Allen (2002 ▶).
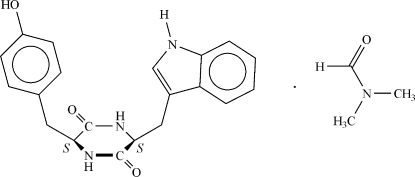


## Experimental

### 

#### Crystal data

C_20_H_19_N_3_O_3_·C_3_H_7_NO
*M* *_r_* = 422.48
Monoclinic, 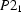

*a* = 6.1923 (2) Å
*b* = 15.3873 (5) Å
*c* = 11.3780 (3) Å
β = 96.661 (1)°
*V* = 1076.81 (6) Å^3^
*Z* = 2
Mo *K*α radiation
μ = 0.09 mm^−1^
*T* = 105 (2) K
0.80 × 0.65 × 0.20 mm

#### Data collection

Siemens SMART CCD diffractometer
Absorption correction: multi-scan (*SADABS*; Sheldrick, 1996 ▶) *T*_min_ = 0.800, *T*_max_ = 0.982
9514 measured reflections
2786 independent reflections
2454 reflections with *I* > 2σ(*I*)
*R*_int_ = 0.038

#### Refinement

*R*[*F* ^2^ > 2σ(*F* ^2^)] = 0.045
*wR*(*F* ^2^) = 0.122
*S* = 1.15
2786 reflections
297 parameters
1 restraint
H atoms treated by a mixture of independent and constrained refinement
Δρ_max_ = 0.23 e Å^−3^
Δρ_min_ = −0.27 e Å^−3^

### 

Data collection: *SMART* (Bruker, 1998 ▶); cell refinement: *SAINT-Plus* (Bruker, 2001 ▶); data reduction: *SAINT-Plus*; program(s) used to solve structure: *SHELXS97* (Sheldrick, 2008 ▶); program(s) used to refine structure: *SHELXTL* (Bruker, 2000 ▶); molecular graphics: *SHELXTL*; software used to prepare material for publication: *SHELXTL*.

## Supplementary Material

Crystal structure: contains datablocks I, global. DOI: 10.1107/S1600536808000640/pv2058sup1.cif

Structure factors: contains datablocks I. DOI: 10.1107/S1600536808000640/pv2058Isup2.hkl

Additional supplementary materials:  crystallographic information; 3D view; checkCIF report

## Figures and Tables

**Table 1:** Hydrogen-bond geometry (Å, °)

*D*—H⋯*A*	*D*—H	H⋯*A*	*D*⋯*A*	*D*—H⋯*A*
N1—H1⋯O1^i^	0.96 (3)	1.94 (3)	2.902 (3)	174 (3)
N2—H2⋯O3^ii^	0.87 (3)	2.01 (3)	2.884 (3)	178 (3)
N3—H3⋯O1^iii^	0.81 (4)	2.16 (4)	2.851 (3)	144 (3)
O2—H4⋯O1*D*	1.04 (5)	1.59 (5)	2.606 (3)	163 (4)

Symmetry codes: (i) 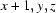; (ii) 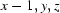; (iii) 
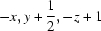
.

**Fig. 1.**
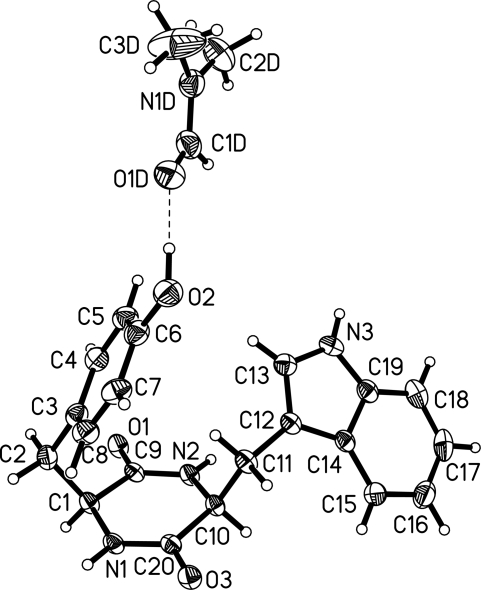
: The molecular structure of (I). Displacement ellipsoids are shown at the 50% probability level.

**Fig. 2.**
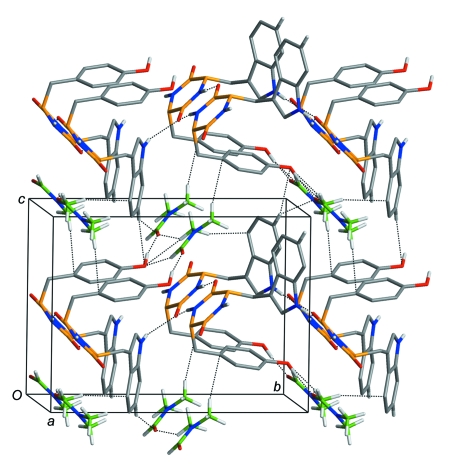
: Crystal packing arrangement viewed approximately along the a axis. Hydrogen bonds and weaker interactions have been indicated by dashed lines; non-essential peptide H atoms have been left out for clarity.

## supplementary crystallographic information

### Comment

The title compound, (3*S*,6S)-3-(4-hydroxsybenzyl)-6 -(1*H*-indol-3-yl)methylpiperazine-2,5-dione dimethylformamide solvate, (I), was obtained as the result of an attempt to crystallize the corresponding linear dipeptide Tyr-Trp from a dimethylformamide solution. In our laboratory we have previously observed similar cyclization reactions taking place for several other dipeptides including Asp-Ala (Görbitz, 1987), Ile-Ile (Görbitz & Hartviksen, 2006) and Val-Leu (unpublished results).

The crystal structure of (I) contains straight hydrogen-bonded tapes as seen in Fig. 2. A search in the Cambridge Structural Database (CSD; Version 5.28 of November 2006; Allen 2002) revealed 59 examples of such tapes (unperturbed *e.g.* by additional cyclic connections between the two C^α^-atoms). The periodicity of this pattern, usually corresponding to a unit-cell parameter, shows a quite narrow distribution with 54 out of 59 observations in the range 6.06 to 6.26 Å, with 6.19 Å observed for (I) being close to the 6.16 Å average value. The full range observed in the CSD is from 6.01 to 6.57 Å, with the upper limit being represented by a distinct outlier (piperazine-2,5-dione:2,5-dihydroxyterephatlic acid 1:1, Luo & Palmore, 2002).

The carbonyl O atom of the cocrystallized dimethylformamide (DMF) solvent molecule accepts a H atom from the Tyr hydroxyl group, but the DMF molecule is also involved in a number of weaker hydrogen bonds (Table 2 and Fig. 2). This is in line with previous findings that DMF molecules are often more heavily involved in intermolecular interactions than one traditionally would expect (Görbitz & Hersleth, 2000).

From the 20 naturally occurring amino acids one can construct 210 different cyclic dipeptides (as opposed to 400 linear dipeptides). Single crystal structural studies have been presented for 20 of them, including four structures with Tyr or Trp residues: *cyclo*(Gly-Trp) (Morris *et al.*, 1974), *cyclo*(Trp-Trp) DMSO solvate (Grant *et al.*, 1999), *cyclo*(Leu-Tyr) hydrate (Suguna *et al.*, 1984), and *cyclo*(Ser-Tyr) hydrate (Lin & Webb, 1973). From a conformational point of view these four peptides resemble (I) as well as each other, and all except *cyclo*(Trp-Trp) crystallize in the *P*2_1_ space group. Nevertheless, each cyclic dipeptide has its own unique packing arrangement, also when all other compounds with a diketopiperazine moiety are considered.

### Experimental

The corresponding linear peptide Tyr-Trp was obtained from Bachem. Crystals of the cyclic analogue resulting from ring closure were obtained by slow evaporation of a solution of Tyr-Trp in dimethylformamide.

### Refinement

Positional parameters were refined for H atoms involved in short hydrogen bonds. Other H atoms were positioned with idealized geometry and fixed C—H distances for CH_3_, CH_2_, CH and aromatic CH type H-atoms at 0.98, 0.99, 1.00 and 0.95 Å, respectively; *U*_iso_ values were 1.2*U*_eq_ of the carrier atom or 1.5*U*_eq_ for the OH and methyl groups. In the absence of significant anomalous scattering effects, 2334 Friedel pairs were merged. The absolute configuration was known for the purchased material.

### Crystal data

**Table:**

C_20_H_19_N_3_O_3_·C_3_H_7_NO	*F*_000_ = 448
*M**_r_* = 422.48	*D*_x_ = 1.303 Mg m^−^^3^
Monoclinic, *P*2_1_	Mo *K*α radiation λ = 0.71073 Å
Hall symbol: P 2yb	Cell parameters from 7819 reflections
*a* = 6.1923 (2) Å	θ = 1.8–28.3º
*b* = 15.3873 (5) Å	µ = 0.09 mm^−^^1^
*c* = 11.3780 (3) Å	*T* = 105 (2) K
β = 96.6610 (10)º	Block, colourless
*V* = 1076.81 (6) Å^3^	0.80 × 0.65 × 0.20 mm
*Z* = 2	

### Data collection

**Table:**

Siemens SMART CCD diffractometer	2786 independent reflections
Radiation source: fine-focus sealed tube	2454 reflections with *I* > 2σ(*I*)
Monochromator: graphite	*R*_int_ = 0.038
Detector resolution: 8.3 pixels mm^-1^	θ_max_ = 28.3º
*T* = 105(2) K	θ_min_ = 1.8º
Sets of exposures each taken over 0.3° ω rotation scans	*h* = −8→8
Absorption correction: multi-scan(SADABS; Sheldrick, 1996)	*k* = −20→20
*T*_min_ = 0.800, *T*_max_ = 0.982	*l* = −12→15
9514 measured reflections	

### Refinement

**Table:**

Refinement on *F*^2^	Secondary atom site location: difference Fourier map
Least-squares matrix: full	Hydrogen site location: inferred from neighbouring sites
*R*[*F*^2^ > 2σ(*F*^2^)] = 0.045	H atoms treated by a mixture of independent and constrained refinement
*wR*(*F*^2^) = 0.122	*w* = 1/[σ^2^(*F*_o_^2^) + (0.0645*P*)^2^ + 0.2252*P*] where *P* = (*F*_o_^2^ + 2*F*_c_^2^)/3
*S* = 1.15	(Δ/σ)_max_ = 0.001
2786 reflections	Δρ_max_ = 0.23 e Å^−^^3^
297 parameters	Δρ_min_ = −0.27 e Å^−^^3^
1 restraint	Extinction correction: none
Primary atom site location: structure-invariant direct methods	

### Special details

**Table:**

Geometry. All e.s.d.'s (except the e.s.d. in the dihedral angle between two l.s. planes) are estimated using the full covariance matrix. The cell e.s.d.'s are taken into account individually in the estimation of e.s.d.'s in distances, angles and torsion angles; correlations between e.s.d.'s in cell parameters are only used when they are defined by crystal symmetry. An approximate (isotropic) treatment of cell e.s.d.'s is used for estimating e.s.d.'s involving l.s. planes.
Refinement. Data were collected by measuring three sets of exposures with the detector set at 2θ = 29°, crystal-to-detector distance 5.00 cm. Refinement of *F*^2^ against ALL reflections.

### Fractional atomic coordinates and isotropic or equivalent isotropic displacement parameters (Å^2^)

**Table:**

	*x*	*y*	*z*	*U*_iso_*/*U*_eq_	
O1	0.1720 (3)	−0.00215 (12)	0.52224 (17)	0.0277 (4)	
O2	0.6882 (4)	0.38722 (14)	0.7195 (2)	0.0378 (5)	
H4	0.566 (7)	0.409 (3)	0.768 (4)	0.057*	
O3	0.8442 (3)	0.11985 (12)	0.31396 (17)	0.0269 (4)	
N1	0.7154 (3)	0.02668 (14)	0.44228 (19)	0.0230 (4)	
H1	0.867 (5)	0.014 (2)	0.465 (3)	0.028*	
N2	0.2984 (3)	0.09051 (13)	0.39160 (19)	0.0223 (4)	
H2	0.162 (6)	0.101 (2)	0.369 (3)	0.027*	
N3	−0.0323 (4)	0.35012 (15)	0.3555 (2)	0.0268 (4)	
H3	−0.123 (6)	0.379 (3)	0.383 (3)	0.032*	
C1	0.5533 (4)	−0.00266 (16)	0.5160 (2)	0.0231 (5)	
H11	0.5496	−0.0676	0.5117	0.028*	
C2	0.6176 (4)	0.02202 (18)	0.6471 (2)	0.0280 (5)	
H21	0.5086	−0.0024	0.6951	0.034*	
H22	0.7596	−0.0050	0.6747	0.034*	
C3	0.6339 (4)	0.11897 (18)	0.6686 (2)	0.0269 (5)	
C4	0.4638 (4)	0.16582 (19)	0.7071 (2)	0.0283 (5)	
H41	0.3344	0.1361	0.7206	0.034*	
C5	0.4777 (4)	0.2550 (2)	0.7265 (2)	0.0317 (6)	
H51	0.3601	0.2854	0.7543	0.038*	
C6	0.6650 (5)	0.29970 (19)	0.7049 (2)	0.0305 (6)	
C7	0.8369 (5)	0.2541 (2)	0.6663 (3)	0.0334 (6)	
H71	0.9651	0.2840	0.6515	0.040*	
C8	0.8222 (4)	0.1643 (2)	0.6491 (2)	0.0307 (6)	
H81	0.9417	0.1336	0.6239	0.037*	
C9	0.3262 (4)	0.02939 (16)	0.4750 (2)	0.0214 (4)	
C10	0.4684 (4)	0.13210 (15)	0.3333 (2)	0.0203 (4)	
H101	0.4283	0.1259	0.2460	0.024*	
C11	0.4861 (4)	0.23024 (15)	0.3614 (2)	0.0245 (5)	
H111	0.5356	0.2378	0.4467	0.029*	
H112	0.5976	0.2561	0.3164	0.029*	
C12	0.2766 (4)	0.27826 (15)	0.3319 (2)	0.0219 (5)	
C13	0.1520 (4)	0.31262 (17)	0.4122 (2)	0.0259 (5)	
H131	0.1882	0.3108	0.4956	0.031*	
C14	0.1618 (4)	0.29527 (15)	0.2171 (2)	0.0236 (5)	
C15	0.2022 (5)	0.27731 (19)	0.1009 (3)	0.0312 (5)	
H151	0.3336	0.2499	0.0857	0.037*	
C16	0.0465 (6)	0.3004 (2)	0.0084 (3)	0.0397 (7)	
H161	0.0713	0.2876	−0.0706	0.048*	
C17	−0.1462 (5)	0.3422 (2)	0.0292 (3)	0.0426 (7)	
H171	−0.2505	0.3565	−0.0359	0.051*	
C18	−0.1877 (5)	0.36285 (19)	0.1422 (3)	0.0367 (6)	
H181	−0.3178	0.3919	0.1561	0.044*	
C19	−0.0318 (4)	0.33969 (16)	0.2361 (2)	0.0270 (5)	
C20	0.6919 (4)	0.09162 (16)	0.3637 (2)	0.0213 (4)	
O1D	0.4352 (4)	0.46196 (17)	0.8569 (2)	0.0448 (5)	
N1D	0.1638 (4)	0.53136 (19)	0.9359 (2)	0.0370 (5)	
C1D	0.2408 (5)	0.4758 (2)	0.8623 (3)	0.0373 (6)	
H1D	0.119 (6)	0.450 (3)	0.804 (3)	0.045*	
C2D	−0.0661 (6)	0.5502 (2)	0.9296 (4)	0.0509 (9)	
H21D	−0.1468	0.5109	0.8726	0.076*	
H22D	−0.0925	0.6104	0.9041	0.076*	
H23D	−0.1147	0.5419	1.0078	0.076*	
C3D	0.3101 (7)	0.5866 (5)	1.0106 (5)	0.094 (2)	
H31D	0.4563	0.5613	1.0191	0.141*	
H32D	0.2590	0.5916	1.0887	0.141*	
H33D	0.3142	0.6444	0.9747	0.141*	

### Atomic displacement parameters (Å^2^)

**Table:**

	*U*^11^	*U*^22^	*U*^33^	*U*^12^	*U*^13^	*U*^23^
O1	0.0200 (8)	0.0271 (9)	0.0367 (10)	0.0003 (7)	0.0066 (7)	0.0090 (7)
O2	0.0475 (12)	0.0301 (10)	0.0363 (11)	−0.0044 (9)	0.0074 (9)	−0.0026 (8)
O3	0.0188 (8)	0.0296 (9)	0.0328 (9)	0.0005 (7)	0.0059 (7)	0.0044 (8)
N1	0.0159 (9)	0.0234 (9)	0.0304 (10)	0.0030 (7)	0.0053 (7)	0.0032 (8)
N2	0.0160 (9)	0.0208 (9)	0.0303 (10)	0.0003 (7)	0.0046 (8)	0.0045 (8)
N3	0.0237 (10)	0.0212 (10)	0.0363 (12)	0.0008 (8)	0.0071 (8)	−0.0046 (9)
C1	0.0193 (10)	0.0197 (10)	0.0310 (12)	0.0015 (9)	0.0051 (9)	0.0049 (9)
C2	0.0252 (11)	0.0301 (13)	0.0286 (12)	0.0026 (10)	0.0022 (9)	0.0064 (10)
C3	0.0248 (11)	0.0320 (13)	0.0237 (11)	−0.0006 (10)	0.0014 (9)	0.0008 (10)
C4	0.0224 (11)	0.0345 (14)	0.0278 (12)	−0.0012 (10)	0.0024 (9)	0.0005 (10)
C5	0.0277 (12)	0.0385 (15)	0.0289 (13)	0.0020 (11)	0.0036 (10)	−0.0025 (11)
C6	0.0339 (14)	0.0320 (14)	0.0251 (12)	−0.0023 (11)	0.0009 (10)	−0.0020 (10)
C7	0.0279 (13)	0.0412 (15)	0.0318 (13)	−0.0059 (11)	0.0072 (10)	−0.0049 (12)
C8	0.0213 (11)	0.0400 (15)	0.0311 (13)	0.0001 (10)	0.0038 (10)	−0.0017 (11)
C9	0.0173 (10)	0.0192 (10)	0.0276 (11)	0.0019 (8)	0.0028 (8)	0.0001 (9)
C10	0.0151 (9)	0.0192 (10)	0.0266 (11)	0.0001 (8)	0.0022 (8)	0.0030 (9)
C11	0.0204 (10)	0.0192 (11)	0.0336 (13)	−0.0017 (8)	0.0027 (9)	0.0014 (9)
C12	0.0197 (10)	0.0172 (10)	0.0292 (12)	−0.0029 (8)	0.0038 (9)	0.0011 (8)
C13	0.0265 (11)	0.0215 (11)	0.0300 (12)	−0.0035 (9)	0.0049 (9)	−0.0011 (9)
C14	0.0243 (11)	0.0170 (11)	0.0296 (12)	−0.0009 (8)	0.0038 (9)	0.0020 (9)
C15	0.0367 (14)	0.0267 (12)	0.0312 (13)	0.0018 (11)	0.0080 (10)	0.0028 (10)
C16	0.0542 (19)	0.0377 (16)	0.0263 (13)	−0.0017 (13)	0.0012 (12)	0.0041 (12)
C17	0.0484 (17)	0.0380 (16)	0.0381 (15)	0.0011 (14)	−0.0087 (13)	0.0143 (13)
C18	0.0311 (13)	0.0295 (14)	0.0477 (17)	0.0020 (11)	−0.0037 (12)	0.0063 (12)
C19	0.0272 (11)	0.0194 (11)	0.0347 (13)	−0.0022 (9)	0.0046 (10)	0.0022 (10)
C20	0.0191 (10)	0.0212 (10)	0.0234 (11)	0.0023 (8)	0.0012 (8)	−0.0019 (8)
O1D	0.0433 (12)	0.0462 (13)	0.0455 (12)	0.0077 (11)	0.0076 (10)	−0.0020 (11)
N1D	0.0350 (12)	0.0415 (13)	0.0339 (12)	0.0042 (11)	0.0012 (10)	0.0027 (11)
C1D	0.0428 (16)	0.0280 (13)	0.0403 (16)	0.0007 (11)	0.0014 (13)	0.0035 (12)
C2D	0.0373 (16)	0.0400 (18)	0.075 (3)	0.0035 (13)	0.0049 (16)	0.0045 (17)
C3D	0.046 (2)	0.145 (6)	0.086 (3)	0.009 (3)	−0.013 (2)	−0.074 (4)

### Geometric parameters (Å, °)

**Table:**

O1—C9	1.247 (3)	C10—C11	1.545 (3)
O2—C6	1.363 (4)	C10—H101	1.0000
O2—H4	1.04 (5)	C11—C12	1.497 (3)
O3—C20	1.234 (3)	C11—H111	0.9900
N1—C20	1.337 (3)	C11—H112	0.9900
N1—C1	1.453 (3)	C12—C13	1.368 (4)
N1—H1	0.96 (3)	C12—C14	1.437 (4)
N2—C9	1.333 (3)	C13—H131	0.9500
N2—C10	1.456 (3)	C14—C15	1.401 (4)
N2—H2	0.87 (3)	C14—C19	1.418 (3)
N3—C19	1.369 (4)	C15—C16	1.389 (4)
N3—C13	1.371 (3)	C15—H151	0.9500
N3—H3	0.81 (4)	C16—C17	1.399 (5)
C1—C9	1.512 (3)	C16—H161	0.9500
C1—C2	1.546 (4)	C17—C18	1.378 (5)
C1—H11	1.0000	C17—H171	0.9500
C2—C3	1.513 (4)	C18—C19	1.400 (4)
C2—H21	0.9900	C18—H181	0.9500
C2—H22	0.9900	O1D—C1D	1.231 (4)
C3—C4	1.389 (4)	N1D—C1D	1.324 (4)
C3—C8	1.397 (4)	N1D—C3D	1.445 (5)
C4—C5	1.391 (4)	N1D—C2D	1.446 (4)
C4—H41	0.9500	C1D—H1D	1.03 (4)
C5—C6	1.394 (4)	C2D—H21D	0.9800
C5—H51	0.9500	C2D—H22D	0.9800
C6—C7	1.388 (4)	C2D—H23D	0.9800
C7—C8	1.397 (4)	C3D—H31D	0.9800
C7—H71	0.9500	C3D—H32D	0.9800
C8—H81	0.9500	C3D—H33D	0.9800
C10—C20	1.520 (3)		
			
C6—O2—H4	108 (3)	C12—C11—H111	108.9
C20—N1—C1	126.1 (2)	C10—C11—H111	108.9
C20—N1—H1	110 (2)	C12—C11—H112	108.9
C1—N1—H1	120 (2)	C10—C11—H112	108.9
C9—N2—C10	126.4 (2)	H111—C11—H112	107.7
C9—N2—H2	112 (2)	C13—C12—C14	106.2 (2)
C10—N2—H2	121 (2)	C13—C12—C11	125.6 (2)
C19—N3—C13	108.7 (2)	C14—C12—C11	128.2 (2)
C19—N3—H3	122 (3)	C12—C13—N3	110.6 (2)
C13—N3—H3	129 (3)	C12—C13—H131	124.7
N1—C1—C9	113.6 (2)	N3—C13—H131	124.7
N1—C1—C2	111.3 (2)	C15—C14—C19	119.0 (2)
C9—C1—C2	110.1 (2)	C15—C14—C12	134.3 (2)
N1—C1—H11	107.2	C19—C14—C12	106.7 (2)
C9—C1—H11	107.2	C16—C15—C14	118.7 (3)
C2—C1—H11	107.2	C16—C15—H151	120.6
C3—C2—C1	113.7 (2)	C14—C15—H151	120.6
C3—C2—H21	108.8	C15—C16—C17	121.3 (3)
C1—C2—H21	108.8	C15—C16—H161	119.3
C3—C2—H22	108.8	C17—C16—H161	119.3
C1—C2—H22	108.8	C18—C17—C16	121.2 (3)
H21—C2—H22	107.7	C18—C17—H171	119.4
C4—C3—C8	117.9 (3)	C16—C17—H171	119.4
C4—C3—C2	121.6 (2)	C17—C18—C19	117.8 (3)
C8—C3—C2	120.5 (2)	C17—C18—H181	121.1
C3—C4—C5	121.8 (3)	C19—C18—H181	121.1
C3—C4—H41	119.1	N3—C19—C18	130.3 (3)
C5—C4—H41	119.1	N3—C19—C14	107.9 (2)
C4—C5—C6	119.7 (3)	C18—C19—C14	121.8 (3)
C4—C5—H51	120.1	O3—C20—N1	122.7 (2)
C6—C5—H51	120.1	O3—C20—C10	118.4 (2)
O2—C6—C7	117.7 (3)	N1—C20—C10	118.9 (2)
O2—C6—C5	123.0 (3)	C1D—N1D—C3D	120.3 (3)
C7—C6—C5	119.4 (3)	C1D—N1D—C2D	121.5 (3)
C6—C7—C8	120.3 (3)	C3D—N1D—C2D	117.3 (3)
C6—C7—H71	119.9	O1D—C1D—N1D	124.7 (3)
C8—C7—H71	119.9	O1D—C1D—H1D	123 (2)
C7—C8—C3	120.9 (3)	N1D—C1D—H1D	112 (2)
C7—C8—H81	119.6	N1D—C2D—H21D	109.5
C3—C8—H81	119.6	N1D—C2D—H22D	109.5
O1—C9—N2	122.6 (2)	H21D—C2D—H22D	109.5
O1—C9—C1	118.1 (2)	N1D—C2D—H23D	109.5
N2—C9—C1	119.3 (2)	H21D—C2D—H23D	109.5
N2—C10—C20	113.8 (2)	H22D—C2D—H23D	109.5
N2—C10—C11	111.9 (2)	N1D—C3D—H31D	109.5
C20—C10—C11	108.22 (19)	N1D—C3D—H32D	109.5
N2—C10—H101	107.5	H31D—C3D—H32D	109.5
C20—C10—H101	107.5	N1D—C3D—H33D	109.5
C11—C10—H101	107.5	H31D—C3D—H33D	109.5
C12—C11—C10	113.42 (19)	H32D—C3D—H33D	109.5
			
N1—C1—C2—C3	62.3 (3)	C14—C12—C13—N3	−0.2 (3)
C1—C2—C3—C4	97.5 (3)	C11—C12—C13—N3	−178.3 (2)
N2—C10—C11—C12	−55.4 (3)	C19—N3—C13—C12	0.7 (3)
C10—C11—C12—C13	109.8 (3)	C13—C12—C14—C15	179.9 (3)
C20—N1—C1—C9	16.1 (4)	C11—C12—C14—C15	−2.1 (4)
C20—N1—C1—C2	−108.9 (3)	C13—C12—C14—C19	−0.4 (3)
C9—C1—C2—C3	−64.6 (3)	C11—C12—C14—C19	177.6 (2)
C1—C2—C3—C8	−82.1 (3)	C19—C14—C15—C16	−2.7 (4)
C8—C3—C4—C5	−0.1 (4)	C12—C14—C15—C16	177.0 (3)
C2—C3—C4—C5	−179.7 (2)	C14—C15—C16—C17	1.1 (4)
C3—C4—C5—C6	1.2 (4)	C15—C16—C17—C18	0.8 (5)
C4—C5—C6—O2	178.6 (3)	C16—C17—C18—C19	−0.8 (5)
C4—C5—C6—C7	−1.2 (4)	C13—N3—C19—C18	176.7 (3)
O2—C6—C7—C8	−179.7 (3)	C13—N3—C19—C14	−1.0 (3)
C5—C6—C7—C8	0.1 (4)	C17—C18—C19—N3	−178.3 (3)
C6—C7—C8—C3	1.0 (4)	C17—C18—C19—C14	−0.9 (4)
C4—C3—C8—C7	−1.0 (4)	C15—C14—C19—N3	−179.4 (2)
C2—C3—C8—C7	178.6 (3)	C12—C14—C19—N3	0.9 (3)
C10—N2—C9—O1	178.7 (2)	C15—C14—C19—C18	2.7 (4)
C10—N2—C9—C1	−0.4 (4)	C12—C14—C19—C18	−177.1 (2)
N1—C1—C9—O1	170.0 (2)	C1—N1—C20—O3	171.3 (2)
C2—C1—C9—O1	−64.4 (3)	C1—N1—C20—C10	−9.2 (4)
N1—C1—C9—N2	−10.8 (3)	N2—C10—C20—O3	176.5 (2)
C2—C1—C9—N2	114.7 (2)	C11—C10—C20—O3	−58.4 (3)
C9—N2—C10—C20	7.7 (3)	N2—C10—C20—N1	−3.0 (3)
C9—N2—C10—C11	−115.4 (3)	C11—C10—C20—N1	122.1 (2)
C20—C10—C11—C12	178.4 (2)	C3D—N1D—C1D—O1D	5.3 (6)
C10—C11—C12—C14	−67.9 (3)	C2D—N1D—C1D—O1D	174.1 (3)

### Hydrogen-bond geometry (Å, °)

**Table:**

*D*—H···*A*	*D*—H	H···*A*	*D*···*A*	*D*—H···*A*
N1—H1···O1^i^	0.96 (3)	1.94 (3)	2.902 (3)	174 (3)
N2—H2···O3^ii^	0.87 (3)	2.01 (3)	2.884 (3)	178 (3)
N3—H3···O1^iii^	0.81 (4)	2.16 (4)	2.851 (3)	144 (3)
O2—H4···O1D	1.04 (5)	1.59 (5)	2.606 (3)	163 (4)
C1D—H1D···O2^ii^	1.03 (4)	2.89 (4)	3.862 (4)	158
C2D—H21D···O1D^ii^	0.98	2.68	3.387 (4)	129
C2D—H21D···O2^ii^	0.98	2.70	3.671 (4)	171
C2D—H22D···C15^iii^	0.98	2.66	3.602 (4)	163
C3D—H32D···C3^iv^	0.98	2.80	3.660 (4)	147

Symmetry codes: (i) *x*+1, *y*, *z*; (ii) *x*−1, *y*, *z*; (iii) −*x*, *y*+1/2, −*z*+1; (iv) −*x*+1, *y*+1/2, −*z*+2.
